# Draft genome sequences of four bacterial isolates from the Indian Fort Nature Preserve in Geneseo, NY

**DOI:** 10.1128/mra.00634-26

**Published:** 2026-09-16

**Authors:** Jacey Cappa, George Privitera, Andrea Rivera, Elizabeth Hutchison

**Affiliations:** 1 SUNY Geneseo Biology Department, Geneseo, New York, USA; Rochester Institute of Technology, Rochester, New York, USA

**Keywords:** bacterial genome, soil microbe, plant microbe, antimicrobial production

## Abstract

We report the genome sequences of four bacterial strains, *Bacillus subtilis*, *Bacillus pumilus*, *Pseudomonas kermanshahensis*, and *Kocuria rhizophila*, isolated from soil or plant material from Geneseo, NY. Bacterial strains were selected based on preliminary, qualitative screening for antimicrobial production via zones of clearing and/or inhibition against lab strains.

## ANNOUNCEMENT

This study focused on the isolation and genome sequencing of bacteria from local Geneseo, NY environments, specifically the Geneseo Indian Fort Nature Preserve. We selected isolates that exhibited zones of clearing on our initial serial dilution plates and/or inhibited growth of lab strains in downstream experiments. The four bacterial strains were isolated from either soil or plant roots, as described in Table 1. For isolates 4.1, 5.2, and 6.24, we resuspended soil in sterile water and performed four 100-fold dilutions; 50 µL of the solution was plated onto Actinomycete Isolation Agar (AIA) or Starch Casein Agar (SCA). For isolate 10.9, we used ethanol to sterilize plant roots, rinsed with water, and ground root tissue with a sterile mortar and pestle. Dilutions and plating were carried out as for soil samples, and all dilution plates were incubated at 30°C for 5–7 days. Isolates were purified by successive streak plating onto tryptic soy agar (TSA) plates. For isolates 4.1, 5.2, and 6.24, DNA was isolated using an NEB Monarch Spin gDNA Extraction Kit (T3010) using the protocol for gram-positive bacteria. DNA quantity and quality were assessed via gel electrophoresis and Nanodrop spectrophotometer. DNA from strain 10.9 was isolated by SeqCenter using a ZymoBIOMICS DNA Miniprep Kit. Briefly, a loopful of cells was transferred aseptically from the agar surface to 750 µL of the lysis solution and resuspended. The standard kit protocol was followed, and DNA was quantified via a Qubit fluorometer.

**TABLE 1:** Genome statistics for the four bacterial strains

Strain	4.1 (*Bacillus subtilis*)	5.2 (*Pseudomonas kermanshahensis*)	6.24 (*Bacillus pumilus*)	10.9 (*Kocuria rhizophila*)
dDDH values from TYGS	90.1% to strain *Bacillus subtilis* ATCC 6051	80.0% to strain *Pseudomonas kermanshahensis* SWRI100	78.8% to strain *Bacillus pumilus* NCTC 10337	83.6% to strain *Kocuria rhizophila* TA68
Source material	Soil on the bank of a pond	Soil next to a riverbank	Soil associated with *Persicaria* roots	Plant roots from genus *Symphyotrichum*
Assembly program for the final genome draft	Shovill	Unicycler	Shovill	Unicycler
# contigs (>500 bp)	15	60	74	21
Assembled genome length (bp)	4,096,723	6,355,659	3,659,739	2,460,731
Coverage	248×	165×	302×	464×
N50	1,047,562	271,348	165,729	731,222
L50	2	8	6	2
GC (%)	43.7	62.6	41.6	70.4
# predicted genes	4,414	5,834	3,880	2,250
BUSCO statistics	Complete: 99.3%; complete and single copy: 99.1%; duplicated: 0.2%; fragmented: 0.7%; missing: 0.0%; *n*: 450	Complete: 99.7%; complete and single copy: 99.5%; duplicated: 0.3%; fragmented: 0.1%; missing: 0.1%; *n*: 782	Complete: 99.6%; complete and single copy: 99.6%; duplicated: 0.0%; fragmented: 0.4%; missing: 0.0%; *n*: 450	Complete: 97.6%; complete and single copy: 97.6%; duplicated: 0.0%; fragmented: 0.6%; missing: 1.9%; *n*: 537

Illumina paired-end library construction and sequencing was carried out by SeqCenter (Pittsburgh, PA). Libraries were made with tagmentation- and PCR-based prep kits using custom IDT unique dual indices. Target insert sizes were 280 bp. Sequencing was performed on a NovaSeq X Plus machine, producing 2 × 151 bp paired-end reads. Multiplexing was removed, quality control was performed, and adapters were trimmed using bcl-convert1 (v4.2.4).

Genome assembly was done using the Galaxy’s open-source suite of tools (1), using default parameters. Quality control was confirmed via FastQC v0.12.1 (2) and trimming carried out with Fastp v1.0.1 (3). Genomes were assembled using both Shovill v1.1.0 (4) and Unicycler v0.5.1 (5), and assembly quality was assessed via Quast v5.3.0 (6), Bandage v0.9.0 (7), and BUSCO v5.8.0 (8). Shovill produced the highest-quality output for genomes 4.1 and 6.24 and Unicycler for genomes 5.2 and 10.9. Finally, genomes were uploaded and annotated via the Rapid Annotations using the Subsystem Technology server (9). Taxonomy for each strain was assigned based on whole-genome data using the Type (Strain) Genome Server (TYGS) tool (10); pairwise dDDH values > 70%, from formula d4, were used to assign species identifications (Table 1).

## Data Availability

Whole genome assemblies from this study have been deposited in NCBI databases under the genome accession numbers JBYBHG000000000, JBYBHI000000000, JBYBHF000000000, JBYBHH000000000, associated with BioProject PRJNA1459477.
